# Distribution patterns and co-occurrence network of eukaryotic algae in different salinity waters of Yuncheng Salt Lake, China

**DOI:** 10.1038/s41598-024-58636-0

**Published:** 2024-04-09

**Authors:** Jing Yang, Chuanxu Wang, Zhuo Wang, Yunjie Li, Huiying Yu, Jia Feng, Shulian Xie, Xin Li

**Affiliations:** 1 Shanxi Key Laboratory of Yuncheng Salt Lake Ecological Protection and Resource Utilization, College of Life Sciences, Yuncheng University, Yuncheng, 044000 China; 2 School of Life Science, Shanxi University, Taiyuan, 030006 China

**Keywords:** Yuncheng Salt Lake, Eukaryotic algae, Salinity, Environmental parameters, Co-occurrence network, Ecology, Environmental sciences, Limnology

## Abstract

The community structure and co-occurrence pattern of eukaryotic algae in Yuncheng Salt Lake were analyzed based on marker gene analysis of the 18S rRNA V4 region to understand the species composition and their synergistic adaptations to the environmental factors in different salinity waters. The results showed indicated that the overall algal composition of Yuncheng Salt Lake showed a Chlorophyta-Pyrrophyta-Bacillariophyta type structure. Chlorophyta showed an absolute advantage in all salinity waters. In addition, Cryptophyta dominated in the least saline waters; Pyrrophyta and Bacillariophyta were the dominant phyla in the waters with salinity ranging from 13.2 to 18%. *Picochlorum*, *Nannochloris*, *Ulva*, and *Tetraselmis* of Chlorophyta, *Biecheleria* and *Oxyrrhis* of Pyrrophyta, *Halamphora*, *Psammothidium*, and *Navicula* of Bacillariophyta, *Guillardia* and *Rhodomonas* of Cryptophyta were not observed in previous surveys of the Yuncheng Salt Lake, suggesting that the algae are undergoing a constant turnover as the water environment of the Salt Lake continues to change. The network diagram demonstrated that the algae were strongly influenced by salinity, NO_3_^−^, and pH, changes in these environmental factors would lead to changes in the algal community structure, thus affecting the stability of the network structure.

## Introduction

Salt Lakes are bodies of saline water with a salt content > 50 g L^−1^, which are formed in natural processes through the continuous addition of complex substances such as salt and geological conditions^1^. These bodies of water contain important chemical components and are an important source of various inorganic salts for industry^2–4^. Salt Lakes are found all over the world, accounting for about 44% of the water volume and 23% of the total surface area of all lakes on Earth^5^. Salt-tolerant microorganisms and algae are well suited to grow in Salt Lake environments due to the inhibition of high salt concentrations^6^. The complexity of these organisms and their unique metabolic functions, together with their salt-tolerant characteristics and the accompanying low-temperature, radiation, and hypoxia resistance, make them unique biological resources for extreme environments, with great potential for application and development.

It has been pointed out that microorganisms are very sensitive to changes in the environment, and the microbial community structure in a particular habitat can reflect the health of that environment from the side. In turn, environmental changes will cause changes in the community structure of microorganisms, which in turn affects the ecological functions of microorganisms such as carbon metabolism, and ultimately affects the structure of the ecosystem^7^. Due to the unique and harsh conditions of salt lakes, microorganisms living in this environment often evolve different strategies to cope with extreme environmental stresses, thus defining the boundaries of what life can survive. As the largest inland lake in Shanxi Province, Yuncheng Salt Lake is an important water body and natural barrier to maintain the local ecological balance. In recent years, with the rapid social and economic development, the mineral resources of the Salt Lake have been over-exploited and the ecological environment has been seriously damaged. Meanwhile, due to the long-term ecological water shortage, the Yuncheng Salt Lake has begun to show a trend of shrinking, which will certainly cause serious damage to the biological populations that maintain the ecological balance.

In response to these phenomena, researchers and scholars have carried out corresponding studies on Yuncheng Salt Lake^6,8^, but most of them focused on microorganisms, while fewer studies were conducted on the algae and the environment of the Salt Lake. Given that the unique salt environment endows Yuncheng Salt Lake with a unique salt-tolerant flora, further studies on the structure of the phytoplankton community in Yuncheng Salt Lake, its species composition, and its environmental drivers are of great significance in exploring the physiological mechanisms of salt algae. In recent years, high-throughput sequencing based on 18S rRNA gene amplicons has been an effective means to study microbial communities in different habitats, and it can respond to a certain extent to changes in microbial community structure, which is not only affected by interactions between species, but also by environmental fluctuations and regional conditions^9,10^. Based on this, this study aims to explore the changes in the community structure of eukaryotic algae in Yuncheng Salt Lake, analyze the distribution patterns and influencing factors of algae in different salinity ranges in the lake, lay a foundation for understanding the status of algal resources and the development of saline algae in the lake, and provide an important theoretical basis for further in-depth understanding of the adaptive mechanisms of algae in extreme environments.

## Result

### Physical and chemical characteristics of water quality

The water quality indicators at each sampling point of Yuncheng Salt Lake are shown in Table 1. The salinity varied from 6.0 to 34.2%, with an average value of 17.9%. According to the changes in salinity, the sampling points can be divided into low salinity (YH1), medium salinity (YH2-YH7), and high salinity (YH8-YH10). One-way ANOVA also showed highly significant differences between the salinities (*P* < 0.01). pH mean was 7.94, which was weakly alkaline overall. TDS, an indicator characterizing the total dissolved solids content of the water body, varied from 57.97 to 346.33 g L^−1^. The lowest salinity sampling point YH1, and the highest salinity sampling point YH8, corresponded to the minimum and maximum values of TN, TDS, sulfide, TP, and NO_3_^−^, respectively, indicating that a certain correlation between salinity and environmental factors such as nitrogen and phosphorus was presented.

**Table 1:** Environmental properties of 10 samples investigated in the present study.

Sample	Salinity	TN (g L^−1^)	pH	TDS (g L^−1^)	Sulfide (g L^−1^)	TP (g L^−1^)	NO_3_^−^ (g L^−1^)
YH1	6.0	5.99	8.27	57.97	6.24	0.14	2.20
YH2	17.0	16.50	8.07	164.00	21.30	0.56	3.43
YH3	13.2	15.67	7.87	123.33	13.73	0.39	3.61
YH4	17.0	18.13	7.93	188.67	21.10	0.32	3.76
YH5	15.5	18.43	7.93	144.00	13.87	0.31	3.92
YH6	18.0	27.00	7.83	172.00	16.83	0.38	5.11
YH7	13.4	16.53	8.07	134.67	13.13	0.29	4.16
YH8	34.2	74.07	7.67	346.33	27.37	1.43	17.10
YH9	22.0	28.57	7.93	224.67	21.80	0.22	5.97
YH10	22.5	33.63	7.83	229.33	27.17	0.35	6.04
Mean ± SD	17.9 ± 7.42	25.45 ± 18.80	7.94 ± 0.16	178.50 ± 77.40	18.25 ± 6.70	0.44 ± 0.36	5.53 ± 4.23

### Eukaryotic algal community structure and driving factors

**Figure 1:**
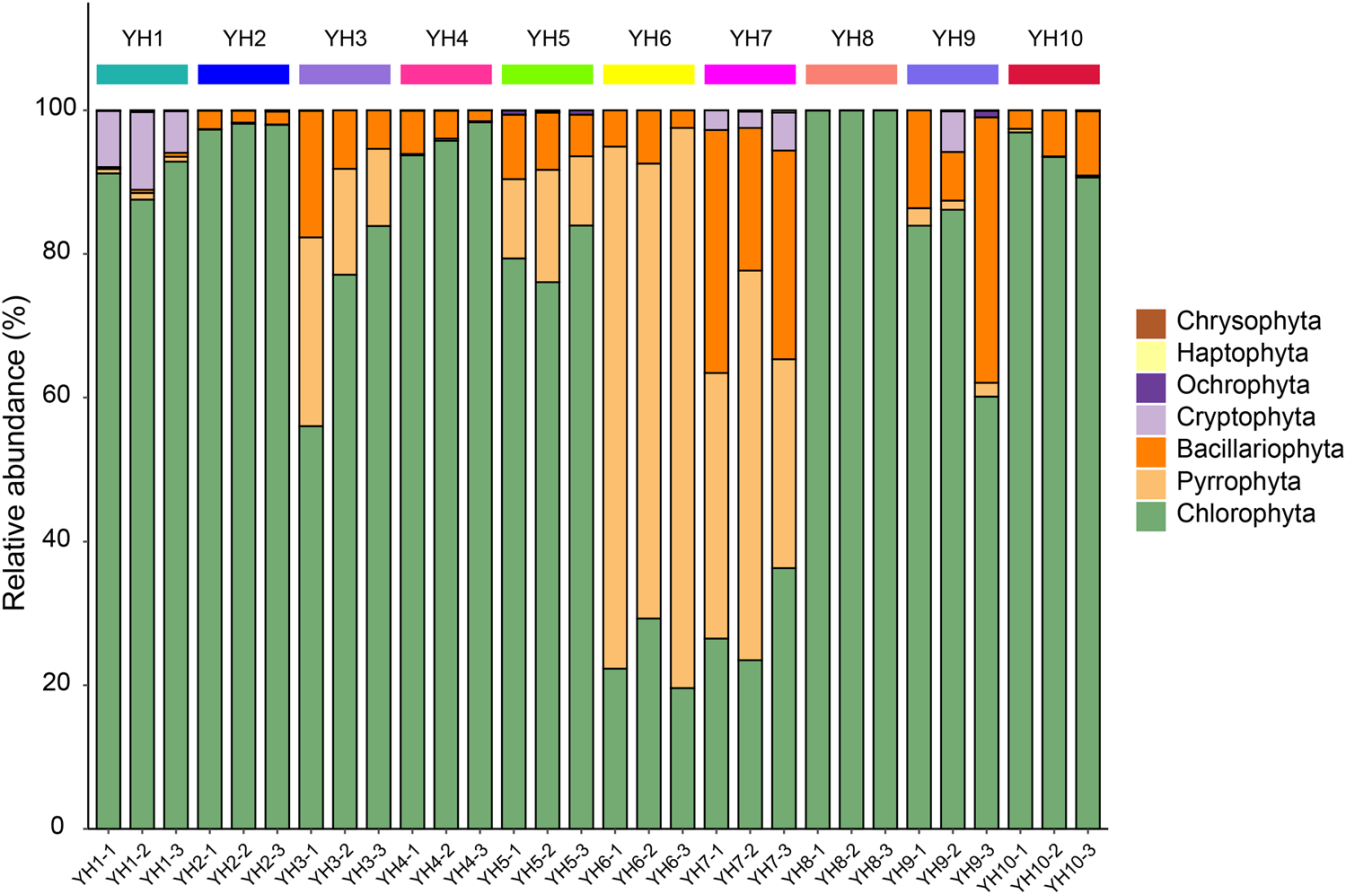
The relative abundance of different phyla in each sample in the present study.

The eukaryotic phytoplankton of Yuncheng Salt Lake is diverse and complex in composition, with a total of 7 phyla, 17 orders, 40 orders, 67 families, 113 genera, and 251 species (estimated) identified. The 7 phylums are Chlorophyta, Bacillariophyta, Pyrrophyta, Cryptophyta, Ochrophyta, Haptophyta, and Chrysophyta. Chlorophyta and Cryptophyta were dominant at the sampling point YH1, which had the lowest salinity. Sampling sites YH3 to YH7 were dominated by Chlorophyta, Pyrrophyta, and Bacillariophyta. In the sampling site YH8 with the highest salinity, the relative abundance of Chlorophyta accounted for more than 99.9%, in addition to less Bacillariophyta and Pyrrophyta (Fig. 1). Therefore, the overall eukaryotic algal composition of Yuncheng Salt Lake showed a Chlorophyta-Pyrrophyta-Bacillariophyta type structure. The diversity index is a measure that reveals the relationship between phytoplankton communities to environmental conditions and the degree of water quality disturbance. For diversity, sampling site YH2 had the highest diversity, and the Shannon index of sampling sites YH6 and YH9 were less than 1, so the waters were heavily polluted (Table 2).

**Table 2:** Statistical results based on amplicon sequencing.

Sample	Ace	Chao	Shannon	Simpson	Coverage
YH1	155.85	154.14	1.26	0.51	1.00
YH2	209.96	207.48	2.14	0.25	1.00
YH3	183.49	182.68	1.45	0.55	1.00
YH4	185.98	182.16	1.77	0.37	1.00
YH5	192.05	191.28	1.92	0.35	1.00
YH6	139.50	137.24	0.57	0.84	1.00
YH7	139.35	137.22	1.07	0.65	1.00
YH8	80.45	79.92	1.25	0.45	1.00
YH9	144.56	143.03	0.72	0.80	1.00
YH10	160.38	159.67	1.64	0.40	1.00

**Figure 2:**
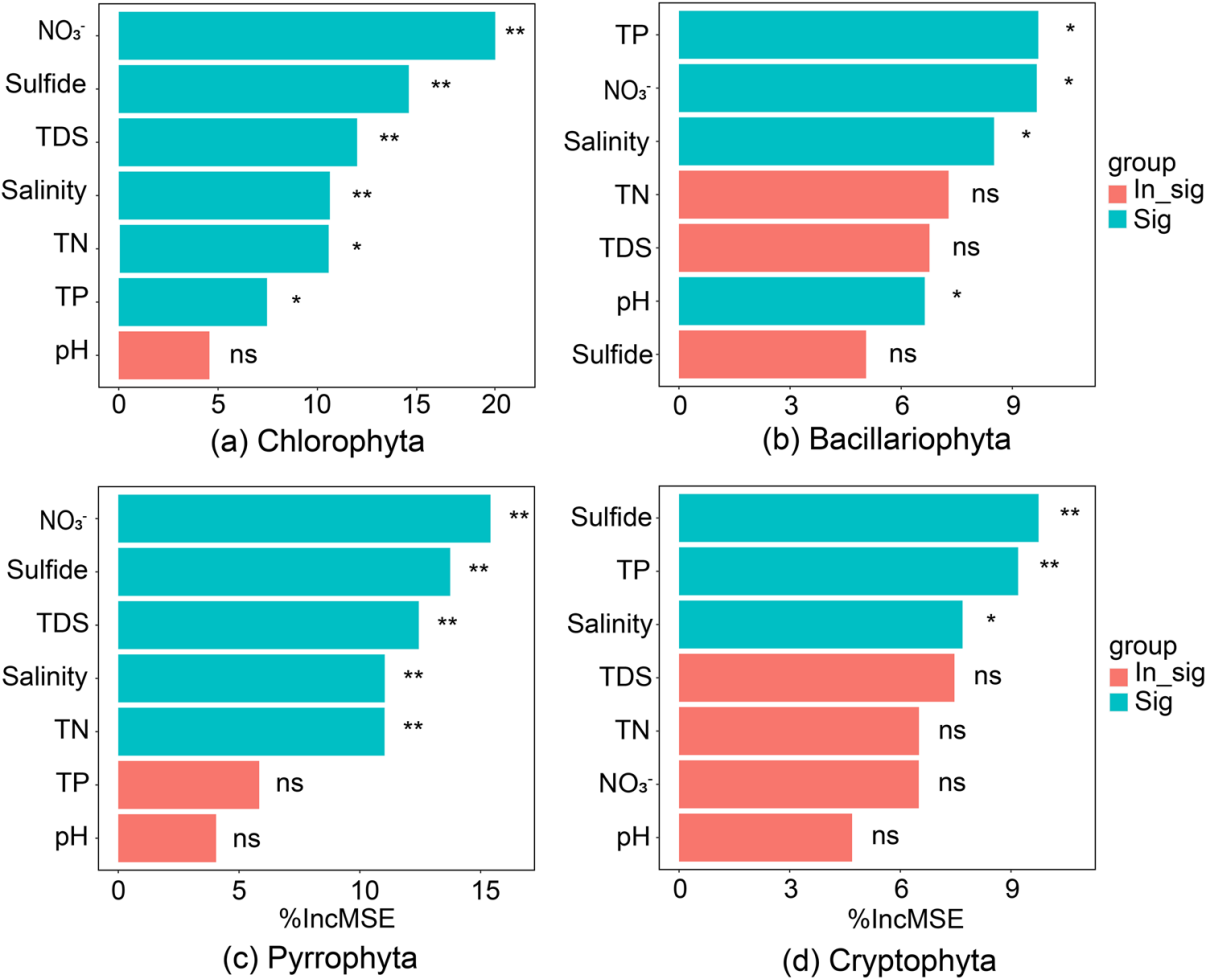
The effects of environmental parameters to phytoplankton based on random forest model.

From the random forest model (Fig. 2), both taxa Chlorophyta and Pyrrophyta were significantly affected by NO_3_^−^, sulfide, TDS, salinity, and TN. The most important variables affecting the abundance of Bacillariophyta were TP, NO_3_^−^, salinity, and pH. Whereas Cryptophyta was mainly affected by sulfide, TP, and salinity. In conclusion, the effect of salinity on all taxa should not be ignored.

### Differences in eukaryotic algal communities analyzed based on LEfSe

**Figure 3:**
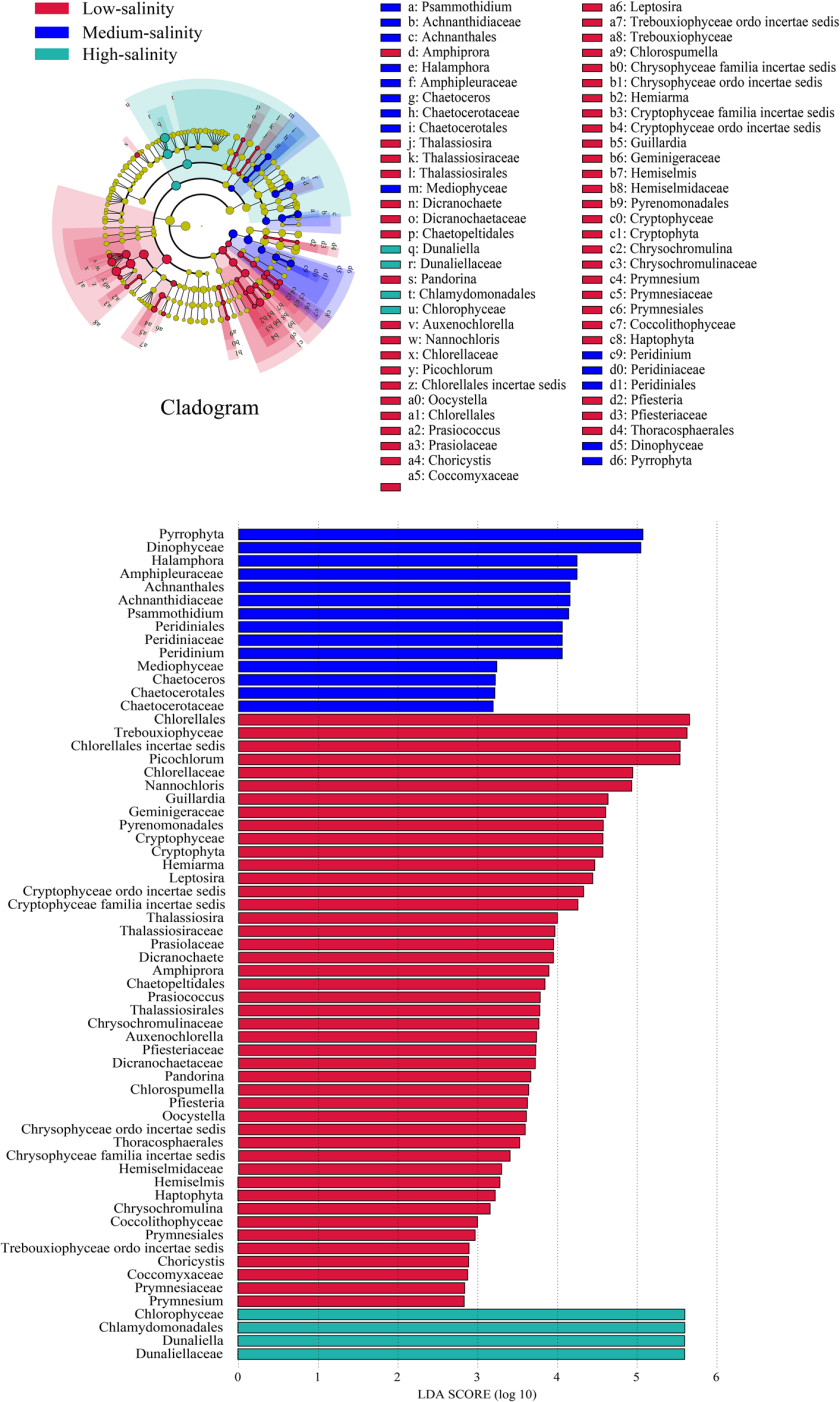
The LEfSe analysis circle dendrogram and histogram of eukaryotic phytoplankton community. The figure is generated using the website http://huttenhower.sph.harvard.edu/lefse/.

To identify taxonomic units that are richly differentiated from phylum to genus level under different salinity conditions, we performed biomarker analysis using the linear discriminant (LEfSe) method (Fig. 3). The results showed that 45 indicator taxa with significant differences were found under low salinity conditions, among which the LDA scores of Chlorellales, Trebouxiophyceae, Chlorellales incertae sedis, *Picochlorum*, Chlorellaceae, *Nannochloris* were relatively high, and were all greater than 4.5. Fourteen taxa were identified as significant at the medium salinity level, namely Pyrrophyta at the phylum level, Dinophyceae at the order level, Amphipleuraceae at the family level, and *Halamphora* at the genus level, in contrast to fewer taxa were identified at the high salinity level with significant differences, including Chlorophyceae, Chlamydomonadales, Dunaliellaceae, *Dunaliella*, with LDA scores greater than 5.0.

**Figure 4:**
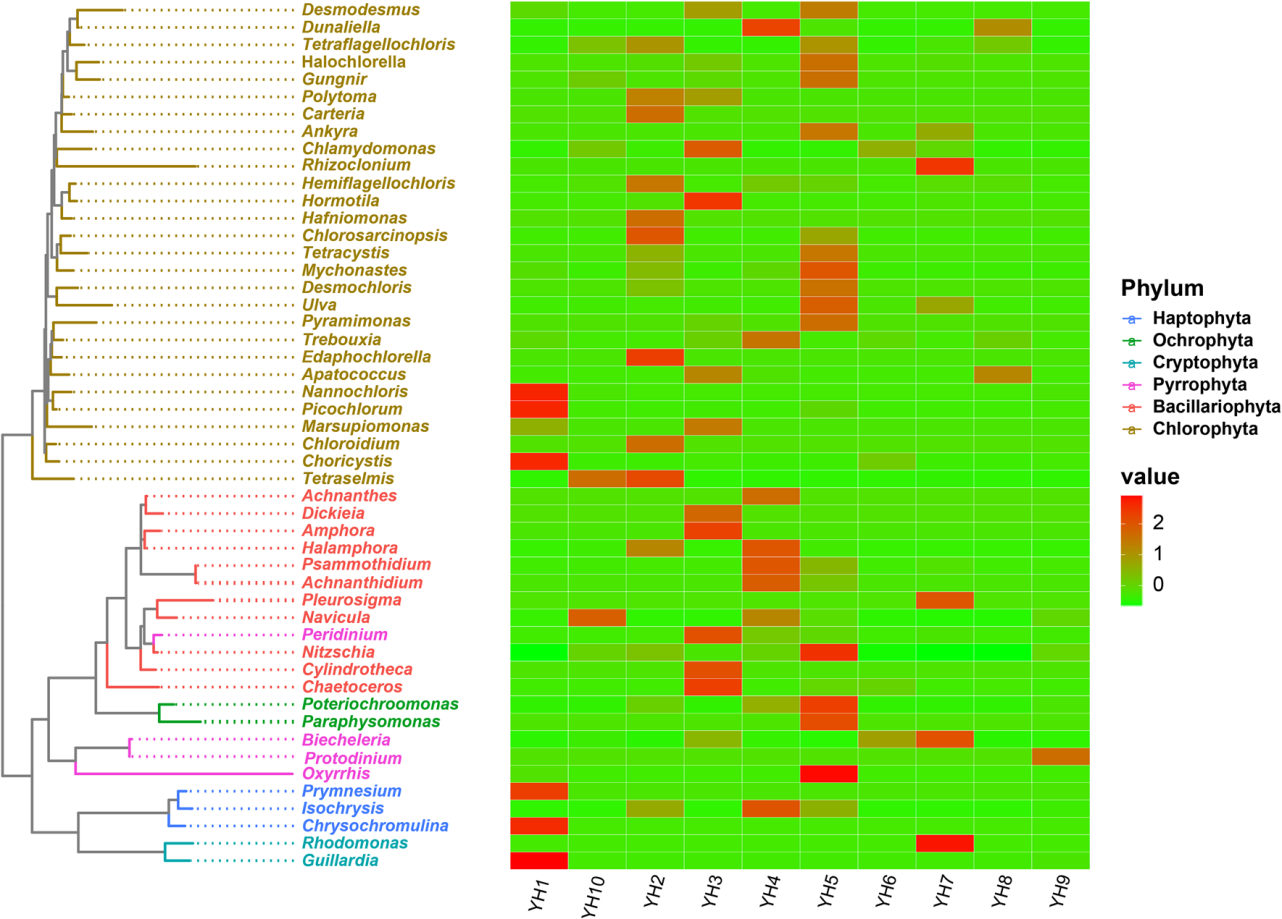
Cluster analysis of algae based on genera level in different regions. The figure is generated using the website https://bioincloud.tech/task-meta.

To identify the most widely distributed genera in salt lakes, we plotted heat maps of the 50 genera with the highest relative abundance (Fig. 4). Results demonstrated that in the low-salinity YH1 sampling site, *Nannochloris*, *Picochlorum*, and *Choricysti*s in Chlorophyta, *Prymnesium* and *Chrysochromulina* in Haptophyta, *Guillardia* in Cryptophyta had high relative abundance. Salt-tolerant alga *Dunaliella* was predominantly distributed at sampling site YH4. *Dickieia*, *Amphora*, *Cylindrotheca*, and *Chaetoceros* in Bacillariophyta were predominantly distributed at sampling site YH3; *Achnanthes*, *Halamphora*, *Psammothidium*, *Achnanthidium*, and *Navicula* were widely distributed in sampling site YH4. The relative abundance of various species of Chlorophyta and Ochrophyta (*Poteriochroomonas* and *Paraphysomonas*) was higher in sampling site YH5. *Dunaliella* and *Apatococcus* are specialized taxa in the most saline sampling site YH8.

### Phylogenetic analysis and distribution characteristics of eukaryotic algal communities

**Figure 5:**
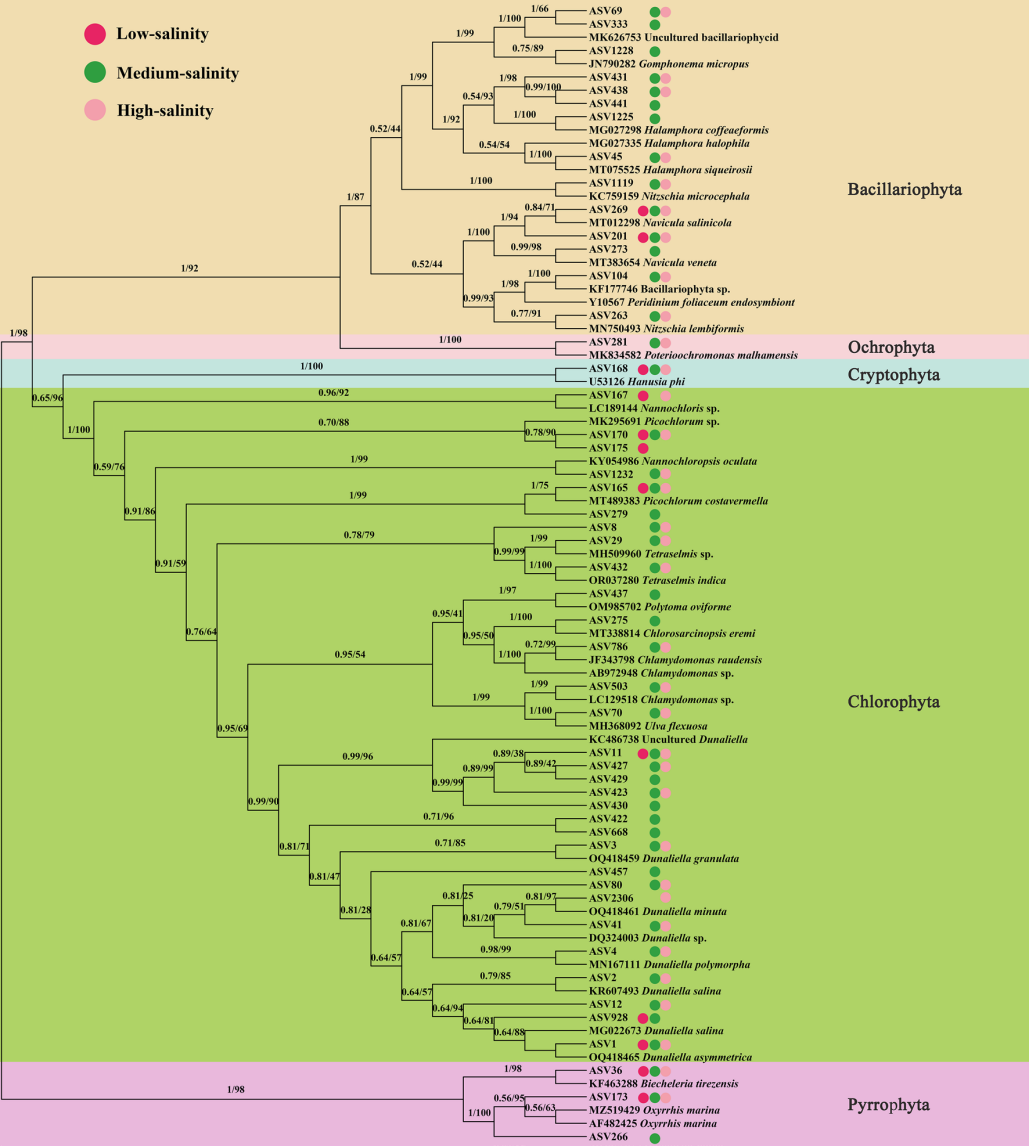
Phylogenetic analysis of the 50 most abundant ASVs. Numbers on the left and right side at the branches represent approximate Bayes test support values and maximum-likelihood bootstrap values. Different color circles followed by the ASVs indicated that the ASV exists in the corresponding community. The figure is generated using the software MAFFT (version 7.394), trimAl (version 1.2), MrBayes (version 3.2.6), and Figtree (version 1.4.2) (http://tree.bio.ed.ac.uk/software/figtree/).

In molecular evolution studies, phylogenetic inference can reveal sequences of biological evolutionary processes, understand the history and mechanisms of biological evolution, and construct evolutionary trees from base differences between sequences at certain taxonomic levels. We first blasted the nt database in NCBI using the 50 most abundant ASVs, downloaded each of the most relevant sequences with the clearest taxonomic annotations, and clustered them with our 50 ASVs, as shown in the phylogenetic tree Fig. 5. In the Chlorophyta, 30 ASVs form a monophyletic evolutionary branch belonging to eight genera. Among them, 16 ASVs formed a monophyletic evolutionary branch belonging to *Dunaliella*, indicating that they contained a richer species composition, mainly including *Dunaliella granulata*, *Dunaliella minuta*, *Dunaliella polymorpha*, *Dunaliella salina*, and *Dunaliella asymmetrica*. However, many ASVs do not have closely related sequences and well-annotated taxonomic information in the nt database, and the taxonomic information supporting them is unclear, forming evolutionary branches that may represent new lineages. Therefore, subsequent isolation, purification, and characterization of the algal communities at the sampling sites are needed to further clarify the taxonomic status of the algae. Fourteen ASVs of diatoms belonging to five genera, of which ASV1225 clustered with *Halamphora coffeaeformi*s, ASV45 with *Halamphora siqueirosii*, and ASV1119 with *Nitzschia microcephala*, respectively, and all had 100% support. ASV168 with *Hanusia phi* in Cryptophyta and ASV281 with *Poterioochromonas malhamensis* in Ochrophyta were clustered into one unit each with 100% support. In Pyrrophyta, 2 ASVs clustered into one unit with *Oxyrrhis marina* and 1 ASV with *Biecheleria tirezensis* with 100 and 98% support, respectively. The distribution of the 50 ASVs is shown in Fig. 5, indicated by circles filled with different colors. The proportions of ASVs distributed in low, medium, and high salinity environments were 24, 94, and 68%, respectively, indicating that most of the taxa in Salt Lake are well adapted to the salinity range between 13.2 and 34%.

### Co-occurrence patterns between eukaryotic algae and physico-chemical parameters

**Figure 6:**
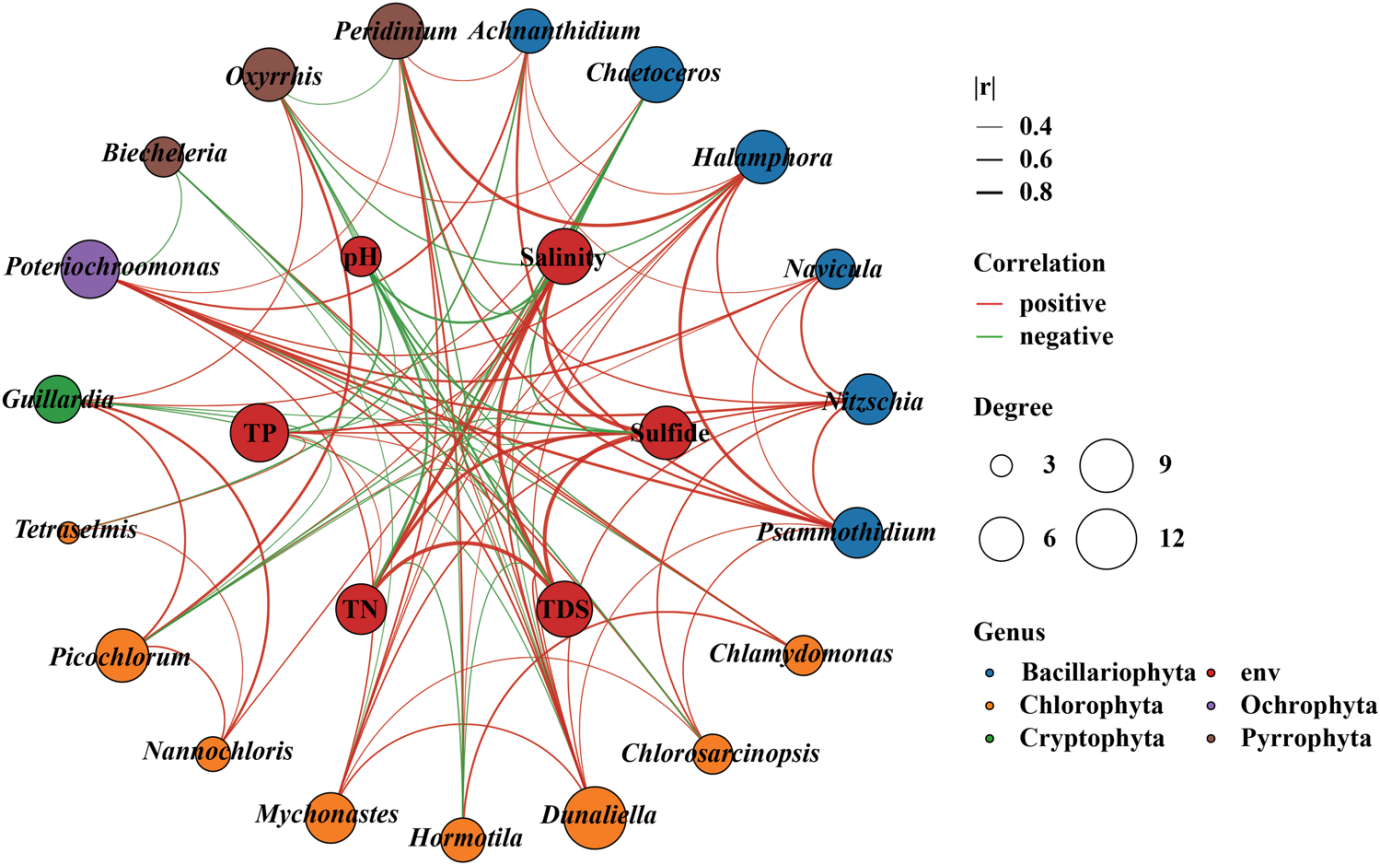
Co-occurrence network between different algal group and environmental parameters. Red lines show positive correlations; green lines show negative correlations. The size of nodes indicates different connectivity degrees. The figure is generated using R software (version 4.0.3) (http://www.R-project.org).

Interactions between different taxa play a crucial role in shaping phytoplankton distribution patterns. They do not exist in isolation in the natural environment, but form a complex network of ecological interactions. Aquatic ecosystems are composed of networks of organisms of different sizes connected by a network of organisms, maintained on the one hand by species interactions (including symbiosis, competition, and parasitism). On the other hand, it is affected by fluctuations in the physico-chemical parameters of the water column. A network diagram (Fig. 6) was constructed by selecting the top 20 genera with high abundance and all the environmental variables to analyze in detail the interspecies interactions between each pair of algae as well as the relationships between algae and environmental variables. The network had 106 edges between each pair of nodes, of which 63.2% were positively connected. Salinity and pH were the key environmental factors that interacted the most in the network, based on their effects on the dominant taxa. *Chaetoceros*, *Dunaliella*, *Picochlorum*, *Poteriochroomonas*, and *Peridinium* were the taxa with the highest degree. *Chaetoceros* was negatively correlated with salinity, sulfide, TDS, and TN, and positively correlated with *Mychonastes*, *Nannochloris*, *Picochlorum*, *Guillardia*, and *Oxyrrhis*, forming a symbiotic relationship. *Dunaliella* was positively correlated with salinity, sulfide, TDS, and TP, tended to coexist with *Mychonastes* and *Poteriochroomonas*, and was mutually exclusive with *Guillardia*, *Biecheleria*, and *Oxyrrhis*.

## Discussion

The Salt Lakes are a special habitat, and the algal distributed in it are bound to have their own special characteristics. However, due to man-made reasons, a series of problems have arisen in the Yuncheng Salt Lake, which have caused the lake to be damaged to different degrees. Around the Salt Lake, some flood control dykes have been damaged to varying degrees, weakening the flood control capacity of the Salt Lake, and in case of flooding, a large amount of mud and stone flow sinks into the Salt Lake, which will affect the balance of the Salt Lake ecosystem. In addition, there are more small enterprises around Salt Lake, and the discharge of industrial wastewater, waste residue, sewage, and garbage from the surrounding residents' lives pollutes the water quality of Salt Lake. All these problems will directly affect the survival of algae in the Salt Lake. Given the current problems faced, an in-depth study of the algae in Yuncheng Salt Lake is very necessary.

The relative abundance of Chlorophyta was observed to be significantly higher than other taxa in this study, contrary to the results observed in many other high-salinity lakes, such as Lake Alchichichica in Mexico^11^, Lake Qarun in Egypt^12^, and Salt Lake in Tibet, China^13^ all had the advantage of diatoms. Besides, the Gahai Salt Lake in the Qaidam Basin of Qinghai, the Xilingol League Salt Lake in Inner Mongolia, and the Salt Lake in Zhangjiakou area are similar to the Yuncheng Salt Lake, which consists of salt ponds with higher salinity and surrounding water bodies with lower salinity^14–16^. The algae in the Yuncheng Salt Lake are more concentrated with the common species in these three Salt Lakes, and show some similarities in algal composition, for example, they all contain some salt-tolerant green algae and diatoms such as *Dunaliella*, *Chlorococcum*, *Navicula*, *Nitzschia*, etc. At the same time, there are also differences between the Salt Lakes, such as *Biecheleria*, *Cryptoperidiniopsoi*, *Oxyrrhis*, *Pfiesteria*, and *Protodinium* of Pyrrophyta; *Chrysochromulina*, *Chlorospumella*, and *Isochrysis* of Chrysophyta; *Guillardia*, *Hemiselmis*, *Hemiarma*, and *Rhodomonas* of Cryptophyta are less reported in other Salt Lakes^16^.

*Dunaliella*, *Picochlorum*, *Nannochloris*, *Chlamydomonas*, *Ulva*, and *Tetraselmis* of Chlorophyta; *Biecheleria*, *Oxyriris*, and *Peridinium* of Pyrrophyta; *Halamphora*, *Psammothidium*, *Navicula*, and *Nitzschia* of Bacillariophyta, and *Guillardia* and *Rhodomona*s of Cryptophyta were the genera with the highest relative abundance in this study. Except for *Dunaliella*, *Chlamydomonas*, *Peridinium*, and *Nitzschia*, the rest of the algal taxa were not observed in the previous surveys of Yuncheng Salt Lake. Thus, it is well illustrated that the algae are undergoing a turnover with the constant changes of the water environment of Salt Lake. It is well known that *Dunaliella* was able to tolerate high salinity, and is an alga with high nutritional value due to the extremely rich carotenoids contained in its cells, which were present and absolutely dominant in all the sampling sites of the Salt Lake. The network diagram showed that *Dunaliella* was strongly influenced by several environmental factors such as salinity, sulfide, TDS, and TP, showing a significant positive correlation (*P* < 0.05). As can be seen in Fig. 5, although more *Dunaliella* were identified in this study, many of them could not be localized to the species level, and it is speculated that many new species may have been generated in the Salt Lake, therefore, based on the principle of distribution of the taxon in the sampling sites in the results, the corresponding samples will be separated and identified to determine the species composition as soon as possible. According to the phylogenetic tree, ASV36 in Pyrrophyta converges with *Biecheleria tirezensis*, with a support rate of 98%. This species was collected in 2017 from a natural pond rich in sulfate in Tirez (Spain)^17^ and has been identified as a new species of euryhaline and eurythermal dinoflagellate, capable of surviving in water bodies with salinity up to 5.6% and temperature ranges of 5–25 °C. As the group with the highest relative abundance at sampling points YH6 and YH7, *Biecheleria* did not find any significant factors affecting its biomass. However, a significant negative correlation was observed with *Poteriochoromonas*, *Myconastes*, *Dunaliella*, and *Chlorosarcinopsis*, indicating that these taxa were mainly influenced by interbiological interactions or is related to other environmental variables not monitored in this study. *Picochlorum* is rich in oil and protein, able to tolerate salt (up to 140 g L^−1^ NaCl) and high temperature (45 °C), and significantly inhibited (*P* < 0.05) by environmental factors such as salinity, sulfide, TDS, TP, NO_3_^−^, whereas mutualistic symbiosis with *Guillardia*, *Oxyrrhis*. In addition, it was shown that species under this taxon such as *Picochlorum atomus* were able to inhibit the growth of the freshwater cyanobacterial pollutant *Pseudanabaena limnetica*^18^, which has a greater application value. Therefore, because of the importance of *Picochlorum* in aquaculture, this species will be isolated, purified, and cultured to further study the effects of salinity and nutrient utilization on this taxon. *Halamphora*, one of the most abundant taxa of Bacillariophyta, is widely distributed at sampling point YH7. Due to its susceptibility to confusion with the *Amphora*, sample collection, isolation, cultivation, and identification will be conducted specifically for this sampling point in the future to further clarify the species composition of this group.

The salinity range of the sample points taken in this study was wide (6.0–34.2%), and it is noteworthy that the salinity of all the sample points exceeded that of seawater (3.5%)^19^, and the salinity of sample point YH8 was as high as 34.2%, which is again an increase from the previously reported data. It has been recorded that the maximum wind force near the Salt Lake can reach 24 m s^−1^, which is equivalent to the wind speed of a class 12 typhoon. Under the influence of such strong wind force, the evaporation of the lake water is more than twice as much as that in other places, and the average annual precipitation in the area is only about one-fourth of the evaporation, which will increase the salinity of the lake water to a certain extent. In addition to this, the high salinity of the surrounding soil also leads to higher salinity in the waters. According to the results of random forest, salinity, NO_3_^−^, and sulfide were the main influences on the distribution of algae. Changes in salinity can lead to the extinction of some species and the emergence of others on the one hand, and on the other hand, it may indirectly lead to food shortages, thus affecting the abundance of plankton^20^. Usually, for very saline water bodies with high osmotic pressure and low dissolved oxygen, conventional algae cannot survive in such extreme environments and only a few species of green algae and diatoms are observed. These algae accumulate macromolecular substances inside the cells, causing an increase in intracellular osmotic pressure to adapt to the high osmotic environment outside. The main species of Bacillariophyta in this type of water were *Navicula* and *Nitzschi*a. This is not only due to the accumulation of macromolecular substances in the cells, but also possibly due to the highly siliceous cell walls on the surface of diatom cells, which can tolerate high osmotic pressure in hypersaline waters. The present study of the relationship between saline algae and the environment enables us to obtain valuable algae from samples more quickly and to obtain pure cultures under experimental conditions for further in-depth research on their specific ecological functions and metabolic mechanisms.

## Conclusion

The eukaryotic phytoplankton of Yuncheng Salt Lake is diverse and more complex in composition, with 7 phyla, 17 classes, 40 orders, 67 families, and 113 genera identified, and the overall structure of Chlorophyta-Pyrrophyta-Bacillariophyta type was presented. There were some differences in the community structure of eukaryotic algae in different salinity waters. Some taxa not observed in previous surveys of the Yuncheng Salt Lake also appeared, such as *Picochlorum*, *Nannochloris*, *Ulva*, and *Tetraselmis* in Chlorophyta, *Biecheleria* and *Oxyrrhi*s in Pyrrophyta, *Halamphora*, *Psammothidium*, and *Navicula* in Bacillariophyta, *Guillardia* and *Rhodomonas* in Cryptophyta. The network diagram indicated that the algal community was strongly influenced by salinity, NO_3_^−^, and pH, changes in these environmental factors will lead to changes in the algal community structure, thereby affecting the stability of the network structure. The results of this study play a very important role in the protection and restoration of biodiversity in Salt Lake, laying a foundation for the subsequent exploration of more salt tolerant algae, and supplementing the germplasm and gene banks of extreme environmental algae.

## Material and methods

### Study area

Yuncheng Salt Lake, also known as the Dead Sea of China, is located in the southern suburb of Yuncheng, Shanxi Province, eastern China (110°7′30″–110°50′00″E, 34°54′00″–35°4′00″N). It is the third-largest natural inland sodium sulfate lake in the world. Yuncheng Salt Lake is rich in manganese, salts, and other substances, and the nearby soil is heavily salinized, with sparse vegetation around it, most of the plants beside the lake are perennial herbaceous plants such as algae and reeds. The concentration of Mg^2+^, Cl^−^, Na^+^, and SO_4_^2−^ in the surface brine is higher than those of other ions, and thus it is considered a typical quaternary salt system of Mg^2+^, Cl^−^, Na^+^, SO_4_^2−^, and H_2_O^6^. It contains important mineral and biological resources. The entire region has a mild climate, belonging to the warm temperate zone.

### Sampling and environmental parameters

**Figure 7:**
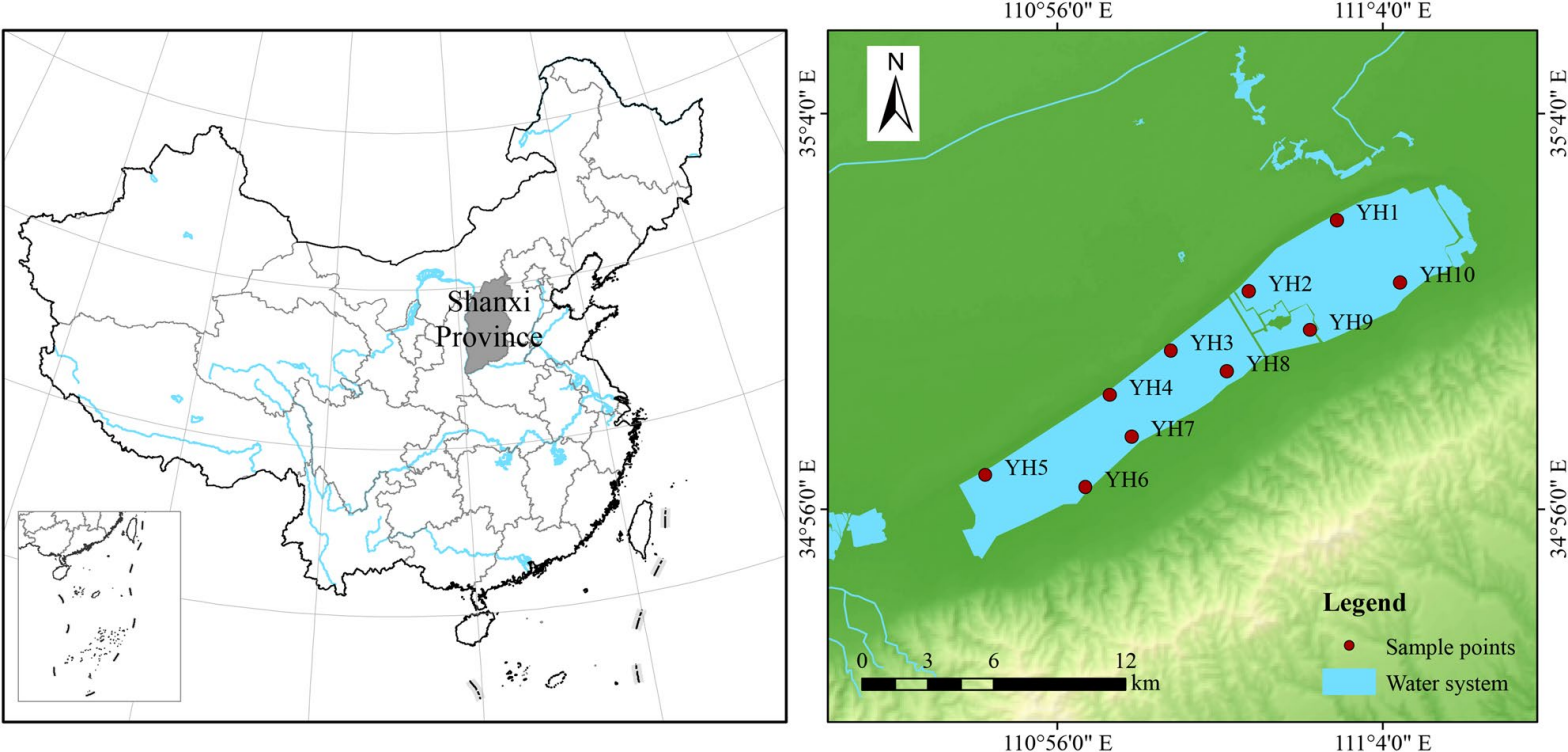
Location of the study area and the sampling points along the Yuncheng Salt Lake. The map is produced using GIS software ArcMap (version 10.2) (https://developers.arcgis.com/).

A total of 30 water samples were collected in April 2023 at 10 sampling sites (Fig. 7). Surface water samples were collected at 0.5 m using a 2 L water picker, and water samples collected in the field were first filtered through a 200 μm sieve silk to eliminate the effects of large zooplankton and particles. A portion of the water samples were then filtered through a 0.45 μm Whatman GF/F membrane, which was folded and placed in a centrifuge tube and stored at − 80 °C for the extraction of environmental genomic DNA, and a portion of the water samples were used for the determination of environmental samples (within 24 h).

Environmental physical and chemical parameters were determined including salinity, pH, total nitrogen (TN), total dissolved solids (TDS), sulfide, total phosphorus (TP), NO_3_^−^. Salinity was determined in situ using a conventional conductivity meter; NO_3_^−^ was determined by UV spectrophotometry; the pH of the lake water was measured with a general portable pH-meter; TN was determined by alkaline potassium persulfate digestion spectrophotometry; TDS was determined by the standard test method for drinking water; Sulfide was determined by inductively coupled plasma emission spectrometry (ICP-AES); and TP was determined by ammonium molybdate spectrophotometry.

### DNA extraction, PCR amplification, and product purification

Genomic DNA was extracted and purified from environmental samples using the E.Z.N.A™ Mag-Bind DNA Kit (OMEGA) kit. The purity and concentration of the DNA extracts were tested using agarose gels to select qualified samples for subsequent analysis. The V4 region of the 18S rDNA gene was amplified using the universal primers V4F (5′-GGGCAAGTCTGGTGCCAG-3′) and V4R (5′-ACGGTATCTRATCRTCTTCTCG-3′). The PCR amplification process consisted of two rounds of amplification: the first round of amplification consisted of 3 min of pre-denaturation at 94 °C; 5 cycles (94 °C denaturation for 30 s, annealing at 45 °C for 20 s, and extension at 65 °C for 30 s); 20 cycles (denaturation at 94 °C for 20 s, annealing at 55 °C for 20 s, and extension at 72 °C for 30 s), and extension at 72 °C for 5 min; and the second round of amplification was pre-denaturation at 95 °C for 3 min, 5 cycles (denaturation at 94 °C for 20 s, annealing at 55 °C for 20 s, and extension at 72 °C for 30 s). And the PCR amplification system consisted of 15 μL of Phusion Master Mix, 1 μL each of 10 μM forward and reverse primers, 10–20 ng of environmental DNA template, and finally double-distilled water (ddH_2_O) to make the total volume of 30 μL.

### Processing sequence data

Raw sequence data were processed using the QIIME 2 software package (version 2019.1)^21^. The DADA2 inference algorithm performs primerless reads to correct sequencing errors and creates amplicon sequence variants (ASVs) for microbial communities^22^. Then, these sequences were quality controlled, denoised, filtered, merged, and chimeras removed to generate ASVs using DADA2. ASVs are more sensitive, specific, and reproducible, and ecological patterns can be better differentiated using ASVs than OTU methods^23^. Raw sequence data were submitted to the NCBI Sequence Read Archive (SRA) database (BioProject accession number PRJNA1013721).

### Statistical analysis

Mothur (http://www.mothur.org) was utilized to calculate α-diversity indices including community diversity (Shannon and Simpson) and community richness (Chao and ACE). Changes in relative abundance of phytoplankton communities at each taxonomic level were plotted by Origin 2018 software.

The random forest (RF) model is a powerful statistical classifier that is well established in other disciplines (e.g., bioinformatics)^24^, but less used in ecology. The model can provide some understanding of the phenomena underlying many problems and characterize the importance (degree of contribution) of some characteristic variables to the classification model^25^. In this study, the random forest model (R 4.0.3) was utilized to assess the degree of influence of environmental parameters on each phytoplankton.

To detect potential biomarkers (biomarkers), Linear discriminant analysis (LDA) effect size (LEfSe) based on normalized relative abundance matrix was used (http://huttenhower.sph.harvard.edu/lefse/). This method first utilizes the Kruskal–Wallis rank sum test to identify characteristics with significant differences in abundance between groups and applies LDA to assess the magnitude of the effect of significantly differing taxa between groups^26^. The default threshold for LDA was 2.0, and the significance level alpha value was 0.05, which was considered statistically significant.

Evolutionary analyses among algae were done online by the platform (https://bioincloud.tech/task-meta). For the phylogenetic analysis, the sequence matrix was first aligned by MAFFT v7.394^27^ with option—auto, and the ambiguously aligned regions were removed using trimAl 1.2^28^ with the option–automated1. Then, IQ-TREE was used to construct the Maximum Likelihood (ML) trees with 5000 ultrafast bootstraps and the Shimodaira-Hasegawa-like approximate likelihood-ratio test^29^. Bayesian phylogenies were inferred using MrBayes 3.2.6. The resulting phylogenetic trees were edited using Figtree 1.4.2 (http://tree.bio.ed.ac.uk/software/figtree/).

To explore the co-occurrence patterns among phytoplankton, network analyses were performed using a correlation matrix, which was constructed by calculating all possible Spearman rank correlations among algae. Spearman correlation between two taxa was considered statistically significant when the Spearman correlation coefficient *R* > 0.8 (or < − 0.8) and *P* < 0.01. Co-occurrence network between different algal groups and environmental parameters in this study was executed in R 4.0.3 software.

## Data Availability

The datasets generated during and/or analysed during the current study are available in the NCBI repository (Accession: SRR25936143, SRR25936140, SRR25936137, SRR25936145, SRR25936142).
